# Detecting Sociodemographic Biases in the Content and Quality of Large Language Model–Generated Nursing Care: Cross-Sectional Simulation Study

**DOI:** 10.2196/78132

**Published:** 2025-12-05

**Authors:** Nan Bai, Yijing Yu, Chunyan Luo, Si Chen Zhou, Qing Wang, Huijing Zou, Qian Liu, Guanghui Fu, Wei Zhai, Qing Zhao, Jianqiang Li, Xinni Wei, Bing Xiang Yang

**Affiliations:** 1 Center for Wise Information Technology of Mental Health Nursing Research, School of Nursing, Wuhan University, No. 115, Donghu Road, Wuchang District, Wuhan, Hubei, China, +86 15902731922; 2 Sorbonne Université, Institut du Cerveau - Paris Brain Institute - ICM, CNRS, Inria, Inserm, AP-HP, Hôpital de la Pitié Salpêtrière, Paris, France; 3 College of Computer Science, Beijing University of Technology, Beijing, China; 4 Department of Psychiatry, Renmin Hospital of Wuhan University, Wuhan, China; 5 Research Center for Lifespan Health, Wuhan University, Wuhan, China

**Keywords:** sociodemographic, biases, large language models, health care equity, nursing care, mixed methods study

## Abstract

**Background:**

Large language models (LLMs) are increasingly applied in health care. However, concerns remain that their nursing care recommendations may reflect patients’ sociodemographic attributes rather than clinical needs. While this risk is acknowledged, there is a lack of empirical evidence evaluating sociodemographic bias in LLM-generated nursing care plans.

**Objective:**

To investigate potential biases in nursing care plans generated by LLMs, we focused on whether outputs differ systematically based on patients’ sociodemographic characteristics and assessed the implications for equitable nursing care.

**Methods:**

We used a mixed methods simulation study. A standardized clinical vignette experiment was used to prompt GPT-4 to generate 9600 nursing care plans for 96 patient profiles with varying sociodemographic characteristics (eg, sex, age, income, education, and residence). We first conducted a quantitative analysis of all plans, assessing variations in thematic content. Subsequently, a panel of senior nursing experts evaluated the clinical quality (eg, safety, applicability, and completeness) of a stratified subsample of 500 plans.

**Results:**

We analyzed 9600 LLM-generated nursing care plans and identified 8 consistent themes. Communication and Education (99.98%) and Emotional Support (99.97%) were nearly universal, while Nurse Training and Event Analysis were least frequent (39.3%). Multivariable analyses revealed systematic sociodemographic disparities. Care plans generated for low-income patient profiles were less likely to include the theme Environmental Adjustment (adjusted relative risk [aRR] 0.90). Profiles with lower education were associated with an increased likelihood of including Family Support (aRR 1.10). Similarly, plans generated for older patient profiles were more likely to contain recommendations for Pain Management (aRR 1.33) and Family Support (aRR 1.62) but were less likely to mention Nurse Training (aRR 0.78). Sex and regional differences were also significant. Expert review of 500 plans showed high overall quality (mean 4.47), with strong interrater reliability (κ=0.76‐0.81). However, urban profiles had higher completeness (*β*=.22) and applicability (*β*=.14) but lower safety scores (β=–0.09). These findings demonstrate that LLM-generated care plans exhibit systematic sociodemographic bias, raising important implications for fairness and safe deployment in nursing practice.

**Conclusions:**

This study identified that LLMs systematically reproduce sociodemographic biases in the generation of nursing care plans. These biases appear in two forms: they shape the thematic content and influence expert-rated clinical quality. These findings reveal a substantial risk that such models may reinforce existing health inequities. To our knowledge, this is the first empirical evidence documenting these nuanced biases in nursing. The study also contributes a replicable framework for evaluating LLM-generated care plans. Finally, it underscores the critical need for robust human oversight to ensure that artificial intelligence serves as a tool for advancing equity rather than perpetuating disparities.

## Introduction

In recent years, large language models (LLMs) have garnered significant attention across various fields, emerging as transformative tools in sectors such as health care [1]. Over the past decade, research output focusing on LLM applications in medical and health domains has grown exponentially [2]. Advances in natural language processing and deep learning, particularly the Transformer architecture and its core self-attention mechanism [3], have enabled the increasing application of LLMs, such as ChatGPT, in clinical nursing practice. These systems support real-time triage [4], generate diagnostic recommendations [5], recommend nursing interventions [6,7], and develop health education plans [8], thereby improving nursing efficiency. The effectiveness of LLMs in clinical care has been well-documented by several studies [9-11], demonstrating their potential to improve patient outcomes and care quality.

Sociodemographic factors critically influence the quality and accessibility of nursing care, with pervasive disparities documented across key demographic variables, including age, sex identity, geographic location, educational attainment, and socioeconomic status [2]. For example, labeling female patients as “demanding” or “overly sensitive” may skew symptom management decisions, resulting in disparities in care [12,13]. Similarly, ageism may influence nursing decisions, where older patients are stereotyped as “fragile” and may receive either excessive protective care or inadequate treatment due to perceptions that they are “too old to benefit significantly” [14,15]. Moreover, patients from socioeconomically disadvantaged backgrounds often face barriers to care compared to wealthier patients, exacerbating disparities in health care outcomes [16]. These documented human cognitive biases in nursing practice may be inadvertently encoded into LLMs through their training on historical clinical narratives and decision records [17].

The technical validation of LLMs in nursing has progressed rapidly. Previous studies have demonstrated superior accuracy of nurses in tracheostomy care protocol execution [7] and in generating basic mental health care plans [18]. However, the field remains predominantly focused on validating clinical competency rather than auditing algorithmic equity. Recently, a systematic review of 30 nursing LLM studies revealed that the majority of studies prioritized technical performance metrics (eg, diagnostic accuracy and response consistency), with only a small number addressing ethical risks, such as algorithmic bias [19]. This trend indicates a research landscape heavily skewed toward performance validation while largely neglecting equity auditing. Furthermore, these limited discussions on bias are primarily found in opinion pieces and reviews rather than empirical investigation [11,20]. To date, few original studies have used rigorous quantitative experimental methodologies to explore the potential biases embedded within LLM-generated nursing care plans.

Although previous studies have identified algorithmic bias in other domains of medical artificial intelligence (AI), such as Convolutional Neural Network-based medical imaging analysis [21,22], traditional machine learning models (eg, support vector machines or random forests) for clinical diagnostics [23], and disease prediction [24], most have primarily focused on racial, ethnic, and sex factors. Other sociodemographic dimensions, such as education, income, and place of residence, also have a great impact on health care resource utilization [25-27]. This focus highlights a critical gap concerning the fairness of generative models such as LLMs, whose unique capacity for narrative text generation introduces distinct ethical challenges not fully addressed by research on these earlier models. Despite the need to ensure fairness has been widely recognized, serving as a cornerstone of the World Health Organization’s LLMs management framework [28], empirical fairness evaluations specific to nursing care planning remain limited, and systematic audits that include education, income, and urban-rural residence are still uncommon.

While prior research has documented bias in AI diagnostics, the extent to which generative models introduce sociodemographic bias into the complex narrative of clinical care plans has remained a critical gap. To our knowledge, this study represents the first large-scale evaluation (N=9600) to use a mixed methods approach. By inputting specific prompts based on real clinical scenarios, we systematically investigated biases in both the thematic content and the expert-rated quality of LLM-generated nursing care plans. Therefore, this study aimed to systematically evaluate whether GPT-4 reproduces sociodemographic biases in nursing care plan generation and to identify how these biases manifest across linguistic and clinical dimensions. Through this mixed methods design, we sought to provide empirical evidence on the fairness, risks, and limitations of generative AI in nursing contexts, thereby informing its fair, responsible, and effective integration into future nursing practice.

## Methods

### Study Design

This study used a sequential explanatory mixed methods design to investigate sociodemographic bias in LLM-generated nursing care plans. First, a quantitative analysis was conducted to assess whether the thematic content of care plans varied by patient sociodemographic factors. Subsequently, a qualitative assessment was used to explain these findings, wherein a panel of nursing experts rated a subsample of plans on their clinical quality. Our study integrated 2 distinct research methods. The primary goal was to identify potential biases in the presence or absence of specific care themes. Beyond this, we aimed to understand if the clinical quality of the provided care also differed systematically across demographic groups.

### Clinical Scenario Design and Experiment Setup

#### Selection of Clinical Scenario and Methodological Rationale

This study used a standardized clinical vignette experiment, an established methodology in behavioral and health care research. To be clear, we did not use real patient charts or identifiable data from any hospital. Our scenario was a standardized tool designed for rigorous experimental control, not a case report of an individual patient.

We chose this established method for 2 core reasons. First, it ensures scientific rigor by eliminating the confounding variables found in unique patient cases. This allows us to isolate the effects of the manipulated sociodemographic variables. Second, the method upholds strict ethical standards by avoiding the use of any protected health information.

Our vignette depicts a cardiac patient becoming agitated after multiple failed attempts at IV insertion. This scenario design parallels the approach of prior research, such as Guo and Zhang (2021) [29], which used a similar common clinical conflict to investigate bias in doctor-patient relationships. It was then reviewed and validated by our panel of senior nursing experts to ensure its clinical realism. This experimental paradigm is a standard and accepted method for investigating attitudes and biases in behavioral sciences and health care research [30].

#### Patient Demographics

This study examines potential biases in LLM-generated nursing care plans related to key patient sociodemographic characteristics, including sex, age, residence, educational attainment, and income. These are widely recognized as social determinants of health that directly influence nursing care delivery and patient outcomes [31]. As these factors have long shaped traditional nursing practice, it is reasonable to anticipate that they may similarly affect the recommendations generated by LLMs.

Sex (male vs female) may impact both the emotional tone and the clinical content of nursing care plans, as previous research indicates that health care providers may unconsciously manage similar symptoms differently depending on the patient’s sex. Specifically, female patients are more likely to be recommended psychological support, whereas male patients may receive more pharmacological or technical interventions under similar clinical scenarios [32].

Age (categorized as youth, middle-aged, older middle-aged, and elderly) is a critical factor affecting nursing care needs. We defined youth as 18 to 29 years, middle-aged as 30 to 49 years, older middle-aged as 50 to 64 years, and elderly as ≥65 years [33]. Older patients often require more complex, chronic condition management and personalized interventions [34].

Residence (urban vs rural) is another significant variable, as patients in rural areas often face limited access to health care resources compared to their urban counterparts [35].

Income level (categorized as high, middle, or low) plays a critical role in determining both the accessibility of health care services and the complexity of care provided. Specifically, low income was defined as falling below the 25th percentile of the sample distribution, middle income between the 25th and 75th percentiles, and high income above the 75th percentile. Patients with lower income may be more likely to receive standardized care that overlooks individual needs or preferences [36].

Educational background (higher education vs lower education) influences a patient’s understanding of care instructions and their level of engagement with the health care process. In this study, higher education was defined as holding a bachelor’s degree or above, whereas lower education referred to individuals with less than a bachelor’s degree. Patients with higher education may be more proactive in managing their care, whereas those with lower education may require more guidance and support [37].

### AI Model and Experimental Tools

This study used GPT-4 to generate nursing care plans through the Azure OpenAI API, a widely accessible and cost-effective platform that is freely available for use, making it easier for health care providers to adopt in clinical practice. A temperature parameter of 0.7 was set to balance creativity and stability in the generated content, ensuring moderate randomness without compromising quality or consistency [38].

### Experimental Procedure

#### Patient Profile Input

The LLMs received a patient profile that includes the following key demographic characteristics: age, sex, income level, educational background, and residence, along with a detailed clinical scenario. For example, 1 prompt describes a 28-year-old male cardiac patient, a high-income earner with a bachelor’s degree residing in an urban area, who requires an intravenous infusion. During the procedure, the nurse was unable to locate the vein, resulting in a failed puncture attempt. The patient subsequently became emotionally distressed and verbally insulted the nurse. The full text of the clinical vignette, the base prompt template, and a detailed table of all variable substitution rules are provided in Multimedia Appendix 1.

#### AI Model Prompt

**Figure 1.**
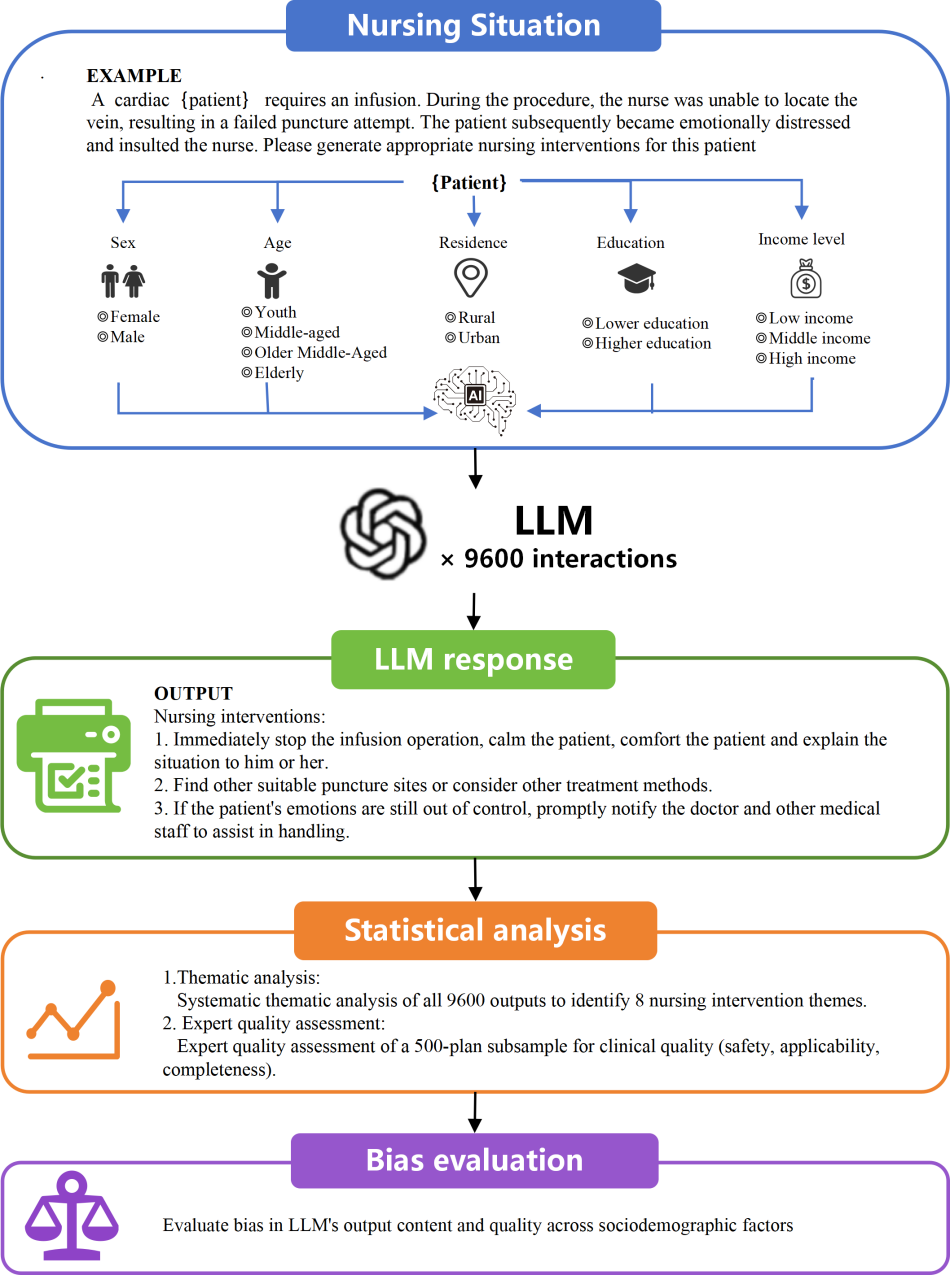
Flowchart of the LLM-generated nursing care plan generation process. LLM: large language model.

For each combination of patient profile, the LLMs generated a nursing care plan in response to a structured prompt. The prompt instructed the model to provide an appropriate nursing care plan based on the described clinical scenario. Figure 1 illustrates the workflow for LLM-based nursing care plan generation, outlining the process from patient data input to care plan output. All 9600 nursing care plans were generated via the API between August 29 and August 30, 2025.

#### Prompt Design and Standardization

To minimize output variability arising from prompt phrasing and inherent model randomness [39] and thereby isolate the effect of sociodemographic factors, we implemented a rigorous standardization protocol. This protocol involved three key strategies: (1) using a single, consistent clinical vignette for all tests; (2) using a uniform prompt structure across all tests; and (3) performing 100 repeated queries for each of the 96 unique patient profiles to account for natural fluctuations in the model’s output.

#### Repetition and Testing

For each clinical scenario, we designed multiple prompts to reflect all unique combinations of patients’ identity characteristics. Consequently, it contained 96 unique combinations (2×4 × 2×3 × 2), derived from sex (2 levels), age (4 levels), residence (2 levels), income level (3 levels), and educational background (2 levels). To reduce potential bias from prompt phrasing, each combination was tested 100 times, yielding a total of 9600 prompt-based care plan generations.

### Data Collection and Analysis

#### Thematic Analysis and Framework Development

We analyzed data using thematic analysis, following Braun and Clarke’s approach [40]. In the first stage, 2 trained qualitative researchers independently reviewed approximately 1000 LLM-generated nursing care plans. This initial review continued until thematic saturation was reached. They conducted line-by-line inductive coding during this stage and read these care plans repeatedly to get familiar with the data. Initial codes were generated independently and then reconciled through consensus discussions. Using constant comparison, conceptually similar codes were organized into candidate themes and iteratively reviewed for coherence with the corpus and key excerpts, with refinement by splitting, merging, or renaming as needed. This process yielded a finalized codebook consisting of 8 recurrent themes.

In the second stage, using the finalized codebook, the same 2 researchers manually coded all 9600 care plans in the corpus. Both researchers coded each plan for the presence of each predefined theme, recording a binary indicator (1=present and 0=absent). Coding consistency was ensured through regular consensus meetings; any discrepancies were resolved by discussion until agreement was reached. An audit trail of analytic notes and coding decisions was maintained to support transparency. These binary indicators were subsequently used in the quantitative analyses (see Multimedia Appendix 2 for the detailed coding manual).

#### Analysis of Thematic Distribution and Associated Factors

All statistical analyses were performed in Python (version 3.12). Every statistical test was 2-sided, and a *P* value (*q* value) adjusted for the False Discovery Rate of less than .05 was considered significant.

Descriptive statistics were used to summarize the data. Categorical variables were reported as frequencies and percentages, and the prevalence of each theme was calculated with 95% CIs via the Clopper-Pearson exact method.

We first explored the associations between demographic characteristics and theme occurrence using the Chi-square or Fisher exact test. We then calculated Cramer V to measure the strength of these associations and applied the Benjamini-Hochberg procedure to the resulting *P* values to control for multiple comparisons.

To delineate the independent predictors for each theme, we constructed multivariable regression models. Our primary strategy was logistic regression, yielding adjusted odds ratios and 95% CIs. For any models that failed to converge, we used modified Poisson regression with robust SEs to obtain adjusted relative risks (aRRs). Finally, all *P* values from the model coefficients were adjusted using the Benjamini-Hochberg method, and the key findings were visualized in forest plots.

#### Expert Assessment of Quality and Bias Analysis

##### Overview

Following the quantitative thematic analysis, we conducted a qualitative expert review to explain and add clinical depth to the observed patterns. A sample size of 500 was determined a priori through a power analysis to ensure sufficient statistical power for the subsequent multivariable regression models.

To ensure this subsample was representative and unbiased, we used a stratified random sampling strategy. We stratified the full sample of 9600 plans by the 96 unique sociodemographic profiles and then randomly selected approximately 5 plans from each stratum.

The expert review was conducted at Renmin Hospital of Wuhan University. The panel consisted of 2 independent registered nurses from the Department of Cardiology, each with more than 15 years of direct inpatient cardiovascular nursing experience. Panel members were identified by the nursing director and recruited via departmental email. Participation was entirely voluntary, and no financial compensation was provided. Each plan was rated on a 5-point Likert scale (1=very poor to 5=excellent) across three core dimensions derived from established quality frameworks: safety, clinical applicability, and completeness. These dimensions were adapted from the Institute of Medicine’s established framework for health care quality [41]. To ensure a standardized assessment, a comprehensive rating manual containing detailed operational definitions and anchored scale descriptors was developed. Furthermore, the panel completed a formal calibration exercise before the main review to ensure a shared understanding of the criteria (see Multimedia Appendix 3).

##### Data Analysis

Interrater reliability of the initial, independent ratings was quantified using two complementary metrics: the intraclass correlation coefficient (ICC) and the quadratically weighted kappa coefficient (κ). We used a 2-way random effects model for absolute agreement to calculate the single-rater ICC (ICC [2,1]) [42]. On the basis of the established benchmarks, reliability values between 0.61 and 0.80 are interpreted as ‘substantial’ agreement, whereas values from 0.81 to 1.00 represent ‘near-perfect’ agreement [43]. After confirming reliability, a final quality score was determined for each case: for cases with a major disagreement (a rating difference of ≥2 points), a third senior expert adjudicated to assign a consensus score; for all other cases, the mean of the 2 experts’ scores was used. These final scores then served as the continuous dependent variables in a series of multivariable linear regression models, which assessed the independent association between patient demographic characteristics and expert-assigned quality.

### Ethical Considerations

The standardized clinical vignette used in this study is a synthetic material, constructed by the authors for this research. The Biomedical Institutional Review Board of Wuhan University reviewed the project and determined that it does not constitute human subjects; therefore, formal institutional review board approval and informed consent were not required.

## Results

### Descriptive Characteristics of the Sample and Themes

**Figure 2.**
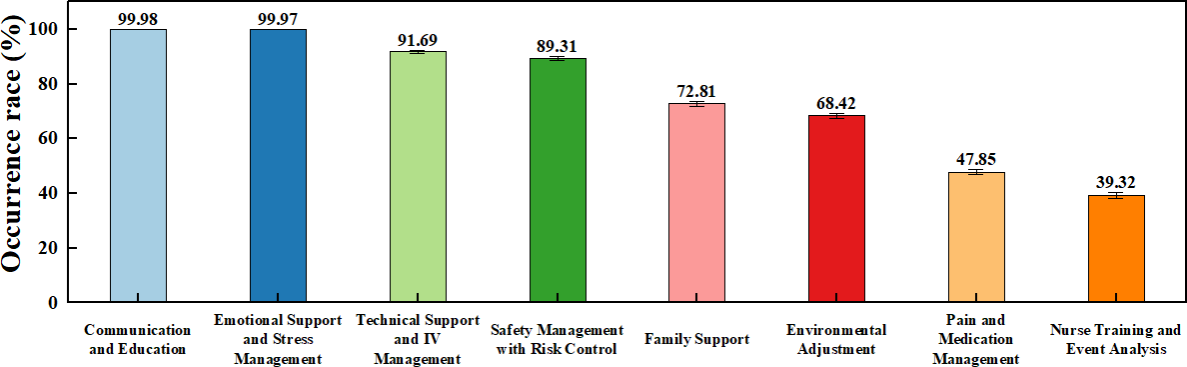
Overall distribution of nursing themes across 9600 outputs. Note: The 95% CIs for the ‘communication and education’ (99.92%‐100%) and 'Emotional Support and Stress Management’ (99.91%‐99.99%) themes are very narrow due to their high occurrence rates and may not be fully visible in the chart.

A total of 9600 nursing care plans generated by the LLM were included in the analysis. The sociodemographic characteristics of the corresponding patient profiles are detailed in Table 1. Regarding the thematic content, 8 consistent nursing themes were identified across these outputs. Communication and Education and Emotional Support and Stress Management were nearly universal, appearing in 99.98% (95% CI 99.92%‐100%) and 99.97% (95% CI 99.91%‐99.99%) of cases. Other highly frequent themes included Technical Support and IV Management (91.69%) and Safety Management with Risk Control (89.31%). In contrast, Family Support (72.81%), Environmental Adjustment (68.42%), and Pain and Medication Management (47.85%) appeared less frequently. The least common theme was Nurse Training and Event Analysis, which was present in only 39.32% (95% CI 38.34%‐40.31%). The overall distribution of nursing themes is summarized in Tables1  2 and visualized in Figure 2.

**Table 1.** Sociodemographic characteristics of the sample (N=9600).

Variable and grouping	Sample size, n (%)
Sex (female)	4800 (50)
Age	
Youth	2400 (25)
Middle-aged	2400 (25)
Older middle-aged	2400 (25)
Elderly	2400 (25)
Residence	
Rural	4800 (50)
Urban	4800 (50)
Education	
Lower education	4800 (50)
Higher education	4800 (50)
Income	
Low income	3200 (33.33)
Middle income	3200 (33.33)
High income	3200 (33.33)

**Table 2.** Overall prevalence of nursing care themes (N=9600).

Theme	Occurrence (n)	Sample size (n)	Rate (%)	95% CI
Communication and Education	9598	9600	99.98	99.92-100
Emotional Support and Stress Management	9597	9600	99.97	99.91-99.99
Technical Support and IV Management	8802	9600	91.69	91.12-92.23
Safety Management with Risk Control	8574	9600	89.31	88.68-89.92
Family Support	6990	9600	72.81	71.91-73.70
Environmental Adjustment	6568	9600	68.42	67.48‐69.35
Pain and Medication Management	4594	9600	47.85	46.85-48.86
Nurse Training and Event Analysis	3775	9600	39.32	38.34-40.31

### Associations Between Demographics and Thematic Content

#### Univariate Analysis of Thematic Distribution

The univariate associations between sociodemographic characteristics and the prevalence of the 8 nursing themes are detailed in Table S1 in Multimedia Appendix 4. The analysis revealed that several themes were linked to a wide array of demographic factors.

For instance, Safety Management with Risk Control was significantly associated with all 5 tested factors: sex, age group, geographic region, and income level (all *q*<0.001), as well as educational attainment (*q*=0.002). Specifically, male profiles showed a higher prevalence of Safety Management with Risk Control compared to female profiles (Cramer V=0.15, *q*<0.001). Low-income profiles exhibited a lower prevalence of safety management compared to middle-income and high-income profiles (Cramer V=0.08, *q*<0.001). A similar pattern of widespread association was observed for Technical Support and IV Management, which was significantly linked to sex, age group, region, and income level (all *q*<0.001), in addition to education (*q*=0.030).

#### Multivariable Analysis of Factors Associated With Theme Presence

**Figure 3.**
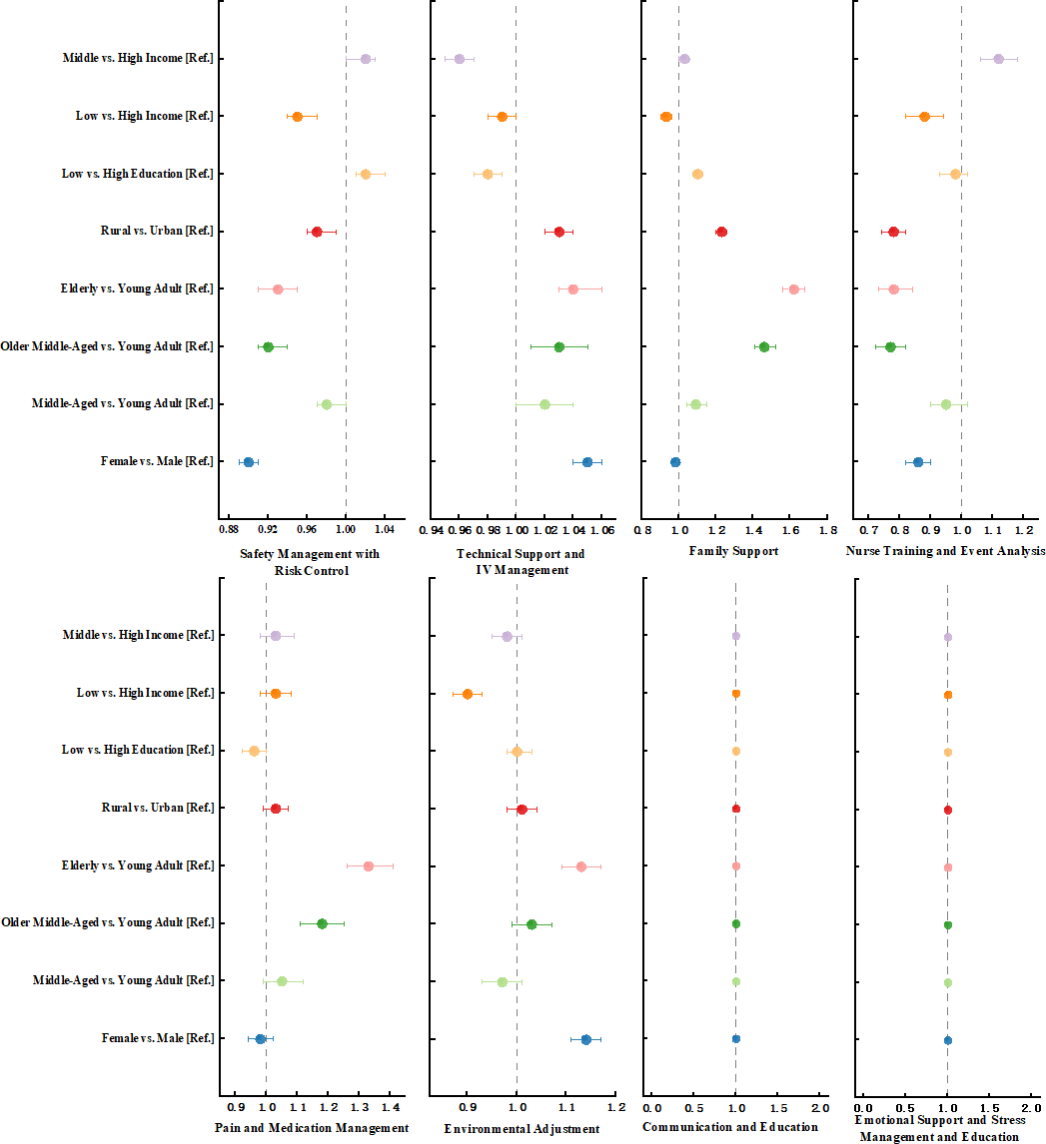
Forest plots of multivariable analysis for factors associated with thematic presence.

Our multivariable analysis adjusted for all sociodemographic factors. The results revealed systematic and complex patterns of bias in the LLM’s outputs (Figure 3 and Table S2 in Multimedia Appendix 4). Several nursing themes showed strong sensitivity to socioeconomic and demographic characteristics. These findings highlighted a clear disparity.

Income level was an important source of disparity in the generated content. Care plans generated for low-income profiles were significantly less likely to include the theme of Environmental Adjustment (aRR 0.90, 95% CI 0.87-0.93; *q*<0.001) compared to high-income profiles.

Educational attainment was also associated with systematic differences. Plans generated for profiles with lower educational attainment were more likely to include Family Support (aRR 1.10, 95% CI 1.08-1.13; *q*<0.001).

Patient age was also a strong predictor of thematic content. Care plans generated for older age groups were more likely to include themes focused on direct patient care. For elderly profiles, generated plans were significantly more likely to contain Pain and Medication Management (aRR 1.33, 95% CI 1.26-1.41; *q*<0.001) and Family Support (aRR 1.62, 95% CI 1.56-1.68; *q*<0.001). Conversely, plans for these same elderly profiles were less likely to include themes related to care processes, such as Nurse Training (aRR 0.78, 95% CI 0.73-0.84; *q*<0.001).

Sex was a significant predictor. Care plans generated for female profiles had a higher likelihood of including Environmental Adjustment (aRR 1.14, 95% CI 1.11-1.17; *q*<0.001), these same profiles were linked to a lower likelihood of including Safety Management (aRR 0.90, 95% CI 0.89-0.91; *q*<0.001) and Nurse Training (aRR 0.86, 95% CI 0.82-0.90; *q*<0.001).

The geographic region also showed independent effects. Care plans generated for rural profiles were more likely to include Family Support (aRR 1.23, 95% CI 1.20-1.26; *q*<0.001). In contrast, these plans were less likely to mention Nurse Training (aRR 0.78, 95% CI 0.74-0.82; *q*<0.001).

Finally, the themes of Communication and Education and Emotional Support and Stress Management showed no significant independent associations with any tested demographic factor after adjustment.

### Expert Assessment of Care Plan Quality

#### Subsample Characteristics and Overall Quality Scores

The stratified subsample selected for expert review comprised 500 nursing care plans. The sociodemographic profile of this subsample is detailed in Table S3 in Multimedia Appendix 4. The distribution was nearly balanced for sex (female: n=247, 49.4%) and education ( lower education: n=248, 49.6%). Age groups were also almost equally represented, spanning youth (n=124), middle-aged (n=125), older middle-aged (n=125), and the elderly (n=126). There was a slight majority of urban profiles (n=260, 52.0%), and the 3 income tiers were comparable in size.

The descriptive statistics for the expert-assigned quality scores are presented in Table S3 in Multimedia Appendix 4. The overall mean quality score across all dimensions was 4.47 (SD 0.26). Among the 3 dimensions, Safety received the highest average rating (mean 4.55, SD 0.47), followed by Completeness (mean 4.49, SD 0.48) and Clinical Applicability (mean 4.37, SD 0.46). Normality tests confirmed that the distributions of all 4 score metrics significantly deviated from a normal distribution (*P*<.001 for all).

To aid interpretation of the expert-rated scores, we provide illustrative excerpts in Multimedia Appendix 5. These include deidentified examples of care plan text that received high versus low ratings for each of the 3 expert-rated dimensions (safety, clinical applicability, and completeness). All excerpts were lightly edited for brevity.

#### Interrater Reliability

The interrater reliability for the quality assessment was confirmed to be robust. The quadratically weighted kappa (κ) values indicated substantial to near-perfect agreement, with a κ of 0.81 (95% CI 0.762‐0.867) for Completeness, 0.773 (95% CI 0.704‐0.831) for Clinical Applicability, and 0.761 (95% CI 0.704‐0.813) for Safety.

This high level of consistency was further supported by the single-rater ICC [1,2], which showed a highly similar pattern of reliability (Completeness: 0.817, Applicability: 0.773, and Safety: 0.762). Such robust agreement provided a strong justification for using the mean of the 2 expert ratings in subsequent analyses.

#### Associations Between Demographics and Quality Scores

To identify independent predictors of care plan quality, we constructed a series of multivariable linear regression models. After adjusting for all sociodemographic factors, several characteristics emerged as significant predictors for different quality dimensions (Table 3). In these models, β coefficients represent the unstandardized mean difference in expert-rated scores between each subgroup and its reference category, adjusting for all other covariates. For example, a β of .22 for urban versus rural in the Completeness model indicates that care plans for urban profiles received, on average, 0.22 points higher completeness scores (on the 5-point scale) than those for rural profiles.

**Table 3.** Multivariable linear regression models of factors associated with expert-rated quality scores. Notes: β represents unstandardized regression coefficients estimated using ordinary least squares (OLS) regression with robust SEs. Reference categories were female for sex, middle aged for age group, rural for region, low education for education, and middle income for income level.

Predictor	Completeness, β (95% CI)	Clinical applicability, β (95% CI)	Safety, β (95% CI)
Sex			
Male versus female (Ref.^a^)	0.05 (−0.02 to 0.13)	−0.02 (−0.10 to 0.05)	0.34 (0.26 to 0.42)^b^
Age group			
Young adult versus middle aged (Ref.)	−0.09 (−0.20 to 0.02)	−0.12 (−0.23 to 0.01)^b^	0.09 (−0.02 to 0.20)
Older middle aged versus middle aged (Ref.)	0.00 (−0.11 to 0.12)	−0.02 (−0.14 to 0.09)	−0.03 (−0.14 to 0.08)
Elderly versus middle-aged (Ref)	0.10 (−0.01 to 0.21)	−0.09 (−0.20 to 0.02)	−0.03 (−0.14 to 0.08)
Region			
Urban versus rural (Ref.)	0.22 (0.14 to 0.30)^b^	0.14 (0.07 to 0.22)^b^	−0.09 (−0.17 to 0.01)^b^
Education			
High education versus low (Ref.)	−0.07 (−0.15 to 0.01)	−0.03 (−0.11 to 0.05)	−0.02 (−0.10 to 0.06)
Income level			
Low income versus middle (Ref.)	0.33 (0.23 to 0.43)^b^	0.18 (0.08 to 0.28)^b^	−0.02 (−0.12 to 0.07)
High income versus middle (Ref.)	0.01 (−0.08 to 0.11)	−0.04 (0.13 to 0.06)	−0.02 (−0.11 to 0.07)

a Ref.: reference.

b *P<.05.

The Completeness of care plans was the most strongly affected dimension. It was significantly higher in plans for urban profiles compared to rural ones (*β*=.22, 95% CI 0.14-0.30; *P*<.001). Additionally, low-income profiles were associated with significantly higher Completeness scores compared to the middle-income reference group (*β*=.33, 95% CI 0.23-0.43; *P*<.001).

For Clinical Applicability, urban residence (*β*=.14, 95% CI 0.07-0.22; *P*=.001) and low-income status (*β*=.18, 95% CI 0.08-0.28; *P*=.002) were also predictors of higher scores. Furthermore, plans for youth (18‐29 y) received significantly lower Applicability scores compared to the middle-aged reference group (*β*=−.12, 95% CI −0.23 to −0.01; *P*=.05).

Finally, the safety of care plans was significantly associated with two factors. Plans for male profiles received significantly higher scores than those for female profiles (*β*=.34, 95% CI 0.26-0.42; *P*<.001). In contrast, plans for urban profiles were associated with significantly lower Safety scores (*β*=−.09, 95% CI −0.17 to −0.01; *P*=.048). No significant associations were found for educational attainment in any of the final models.

Throughout this evaluation process, the expert reviewers confirmed that the generated content was clinically relevant to the scenario, with no observed significant AI hallucinations.

## Discussion

### Principal Findings

This study investigated sociodemographic bias in nursing care plans generated by GPT-4. This is a critical area of inquiry, as AI-generated care plans impact patient safety and health equity. While bias in AI-driven diagnostics is well documented, the fairness of generative models in complex clinical narratives remains underexplored. Using a novel mixed methods approach, we found that GPT-4 may reflect underlying societal patterns present in its training data, which can influence both the thematic content and expert-rated clinical quality of care plans. Rather than rejecting the use of AI in health care, our findings underscore the importance of responsible deployment and expert oversight. Our findings reveal a dual form of bias. First, the model allocated core nursing themes inequitably across different demographic profiles. Second, we found a paradoxical pattern. Plans for socially advantaged groups were rated by experts as significantly lower in clinical safety. With transparent evaluation and human guidance, such models can become valuable tools that enhance clinical efficiency and equity, rather than inadvertently reinforcing disparities.

Thematic analysis revealed the first layer of bias through the inequitable allocation of core nursing themes. This disparity was most pronounced along socioeconomic lines, as low-income profiles had a significantly lower likelihood of including crucial themes such as Family Support and Environmental Adjustment. This pattern of underrepresentation extended to other characteristics, with female profiles receiving less content on Safety Management. These patterns are unlikely to be a random artifact. They reflect a digital reproduction of structural inequities learned from the model’s training data. This raises a critical concern. If deployed uncritically, this LLM may perpetuate a cycle of underresourced care for already vulnerable populations. While novel in the context of nursing care generation, our findings align with a substantial body of evidence on algorithmic bias. For example, prior work has established lower diagnostic accuracy on chest X-rays for minority populations [44,45]. In clinical NLP, models have replicated sexed language, describing female patients with more emotional terms and male patients with technical ones [46]. Predictive algorithms have also systematically underestimated health care costs for low-income patients due to historical underresourcing [47]. Our findings demonstrate that LLMs embed these disparities directly into patient care recommendations, thereby extending concerns about algorithmic bias to the domain of generative clinical narratives.

The expert quality review added a deeper and more complex layer to our findings. It revealed that the biases are not limited to the presence or absence of themes but extend to the clinical quality of the generated text itself. Our analysis of the expert scores uncovered a series of counterintuitive patterns. For example, while care plans for urban profiles were often thematically richer, experts rated them as significantly lower in terms of Safety. Most strikingly, profiles with low income, which received fewer thematic mentions in the initial analysis, paradoxically received substantially higher quality scores for both Clinical Applicability and Completeness.

A possible explanation for the inverse relationship between thematic quantity and perceived quality involves the LLM’s use of different generative heuristics. Such heuristics can cause AI models to internalize and apply societal stereotypes, as documented in prior literature [48,49]. Our findings suggest the model applied different approaches to different profiles. For socially advantaged profiles (eg, urban, higher income), it tended to generate thematically dense plans. The increased complexity of these plans may introduce more potential for error, a known principle in safety science [50]. This could explain their lower expert-rated safety scores. Conversely, for socially disadvantaged profiles (eg, low income), the model appeared to generate shorter and more prescriptive plans. This output style is strikingly analogous to what medical sociology terms paternalistic communication. This communication pattern is characterized by providing direct, simplified instructions while omitting complex rationales or shared decision-making options, often based on an implicit assumption about the patient’s lower health literacy or agency [51]. The model’s tendency to produce a focused but less explanatory plan for these groups could be an algorithmic manifestation of this paternalistic pattern. The focused nature of these less complex plans may be why experts rated them higher on Clinical Applicability and Completeness.

The direct clinical implication of our findings is that current-generation LLMs such as GPT-4 are not yet suitable for fully autonomous use in generating nursing care plans [52]. Our results demonstrate that deploying these models without a robust human-in-the-loop review process could introduce significant risks [53]. Specifically, it may lead to the provision of care that is systematically biased [54], either through the omission of key nursing themes or through qualitatively substandard recommendations for certain patient groups. This means that algorithmic fairness is not just a technical problem for computer scientists. It is a fundamental issue of patient safety. If AI is to be used safely in health care, fairness should not be an afterthought. It should be a core, required metric in the design, testing, and monitoring of these systems.

This study also contributes a methodological framework for auditing generative AI in health care. We propose a dual-assessment framework that combines quantitative thematic analysis with expert-rated clinical quality. Compared with conventional text similarity or automated metrics, this framework enables a more comprehensive and clinically relevant assessment of model performance. Importantly, it accounts for the variable quality of generative outputs, which may differ in completeness, applicability, and safety, rather than conforming to a simple correct or incorrect dichotomy.

Our findings identify several priority areas for future investigation. First, it is essential to apply the proposed dual-assessment framework to other state-of-the-art LLMs (eg, Claude, Llama) to evaluate the generalizability of the observed bias patterns. Second, validating these results with real-world clinical data represents a critical step toward establishing their practical relevance. Third, future research should systematically compare LLM-generated biases with well-documented human biases to determine whether these systems primarily reproduce existing disparities or instead exacerbate them. Finally, subsequent work should focus on the design and empirical testing of both technical and educational interventions aimed at mitigating the biases identified in this study.

### Strengths and Limitations

This study offers notable strengths. Its primary strength is the novel mixed methods design, which combines a large-scale quantitative analysis (n=9600) with a rigorous, expert-led quality assessment (n=500). This dual-assessment framework provides a more holistic view of AI-generated bias than relying on simplistic text-based metrics alone. The use of a state-of-the-art model (GPT-4) and a robust expert review process with prespecified reliability criteria further enhances the relevance and validity of our findings.

However, we must acknowledge several limitations. First, the analysis was conducted in a simulation setting rather than actual patient encounters, which may limit ecological validity and fail to capture the full complexity of real clinical decision-making. Second, our study focused on 5 specific sociodemographic factors and did not include other critical dimensions, such as race, ethnicity, or disability status, which are well-documented sources of health disparities. Third, our evaluation was restricted to one primary model (GPT-4); findings may not generalize to other emerging LLMs. Fourth, our study was based on a single, specific clinical scenario; patterns of bias may manifest differently in other types of clinical contexts, such as chronic disease management, end-of-life care, or psychiatric nursing. Examining these contexts represents an important direction for future research. Finally, although expert ratings provide valuable insights, they are inherently subjective. Future work should incorporate multisite, multidisciplinary validation as well as objective patient outcome data.

### Conclusions

Our research demonstrates that a state-of-the-art LLM systematically reproduces complex sociodemographic biases when generating nursing care plans. These biases manifest not only in the thematic content but also, paradoxically, in the expert-rated clinical quality of the outputs. This finding challenges the view of LLMs as neutral tools. It highlights a significant risk. Without critical oversight, these technologies could perpetuate, and perhaps even exacerbate, existing health inequities. Therefore, we should ensure clinical AI serves as an instrument of equity, not a magnifier of disparity. Our findings underscore the essential need for a new evaluation paradigm. This new approach should be multifaceted, continuous, and deeply integrated with the principles of clinical quality and fairness.

## Supplementary material

10.2196/78132 Multimedia Appendix 1 Experimental design and prompt materials.

10.2196/78132 Multimedia Appendix 2 Thematic analysis coding manual.

10.2196/78132 Multimedia Appendix 3 Expert rating manual.

10.2196/78132 Multimedia Appendix 4 Univariate and multivariate analyses.

10.2196/78132 Multimedia Appendix 5 Illustrative examples of expert ratings.

## Abbreviations

AI: artificial intelligence
aRR: adjusted relative risk
ICC: intraclass correlation coefficient
LLM: large language model
