# Supplementary material for: Detecting Sociodemographic Biases in the Content and Quality of Large Language Model–Generated Nursing Care: Cross-Sectional Simulation Study
Source: J Med Internet Res. 2025 Dec 5;27:e78132. doi: 10.2196/78132 (PMC12683325; doi:10.2196/78132)
Supplement: Multimedia Appendix 2 [file jmir-v27-e78132-s002.docx]

**Multimedia Appendix 2. Thematic Analysis Coding Manual**

| Theme | Operational Definition | Example Quotations |
| --- | --- | --- |
| Theme 1: Communication and Education | Strategies used by nurses to explain procedures, provide health education, and enhance patients’ understanding of their condition and care. | - “Explain the procedure step by step before venipuncture to reduce the patient’s fear.”  - “Use simple and easy-to-understand language, avoiding overly technical terms.”  - “Provide written materials or visual aids to help the patient understand complex information.”  - “Educate the patient about heart disease management to improve adherence.” |
| Theme 2: Emotional Support and Stress Management | Aimed at alleviating patients’ anxiety, fear, and stress, and enhancing coping ability. | - “Listen to the patient’s concerns and provide reassurance with empathy.”  - “Offer psychological counseling or relaxation techniques such as deep breathing.”  - “Encourage the presence of family members to provide emotional support.”  - “Acknowledge the patient’s distress and validate their feelings.”  - “Create opportunities for patients to express emotions and fears openly.” |
| Theme 3: Technical Support and IV Management | Relating to clinical skills, IV therapy, and technical monitoring tasks. | - “Arrange for an experienced nurse or specialist to perform venipuncture.”  - “Consider ultrasound-guided venipuncture to improve success rate.”  - “Use local anesthetics before venipuncture to reduce pain.”  - “Employ vein visualization devices to minimize repeated attempts.”  - “Provide continuous monitoring of cardiac conditions alongside IV procedures.” |
| Theme 4:  Safety Management with Risk Control | Measures to ensure patient and staff safety, prevent violence, and minimize clinical risks. | - “Assess patient’s risk of aggressive behavior and ensure safety measures are in place.”  - “Separate patient from others if necessary to prevent further incidents.”  - “Request security assistance when handling potentially violent situations.”  - “Ensure the clinical environment is safe for staff and patients.”  - “Implement protective restraints following hospital policies when required, while explaining procedures to the patient.” |
| Theme 5:  Family Support | Engagement of family members in patient care to provide emotional, social, and practical support. | - “Encourage family members to participate in treatment and care planning.”  - “Educate family on monitoring patient’s health indicators at home.”  - “Include family in discussions about emotional support strategies.”  - “Guide caregivers on stress management techniques to assist the patient.”  - “Provide information to family members about community resources and social services.” |
| Theme 6: Environmental Adjustment | Modifications of the physical or social environment to facilitate patient comfort, rest, and recovery. | - “Ensure a quiet and comfortable environment to reduce stress.”  - “Adjust lighting and room temperature to enhance comfort.”  - “Reduce environmental triggers that may cause agitation or anxiety.”  - “Provide soothing background music or a calm atmosphere.”  - “Reorganize the ward layout to improve accessibility and mobility.” |
| Theme 7:  Pain and Medication Management | Reflecting pharmacological and non-pharmacological pain relief and medication monitoring | - “Provide relaxation or distraction techniques to manage pain.”  - “Use local anesthetics before venipuncture to minimize discomfort.”  - “Monitor for side effects of medications and adjust treatment accordingly.”  - “Educate patients on proper medication use and potential reactions.” |
| Theme 8:  Nurse Training and Event Analysis | Professional development, staff training, and analysis of incidents to improve patient care. | - “Provide workshops and peer-learning sessions to improve clinical skills.”  - “Document patient incidents thoroughly for future reference and staff training.” |
