# Supplementary material for: Detecting Sociodemographic Biases in the Content and Quality of Large Language Model–Generated Nursing Care: Cross-Sectional Simulation Study
Source: J Med Internet Res. 2025 Dec 5;27:e78132. doi: 10.2196/78132 (PMC12683325; doi:10.2196/78132)
Supplement: Multimedia Appendix 4 [file jmir-v27-e78132-s004.docx]

**Supplementary Table S1. Univariate Analysis of the Association Between Sociodemographic Characteristics and Thematic Presence**

| Characteristic | Theme 1： Communication and Education (%) | Theme 2： Emotional Support and Stress Management (%) | Theme 3：Technical Support and IV Management (%) | Theme 4： Safety Management with Risk Control (%) | Theme 5： Family Support (%) | Theme 6：Environmental Adjustment (%) | Theme 7： Pain and Medication Management (%) | Theme 8： Nurse Training and Event Analysis (%) |
| --- | --- | --- | --- | --- | --- | --- | --- | --- |
| All Patients | 99.98 | 99.97 | 91.68 | 89.31 | 72.82 | 68.41 | 47.85 | 39.32 |
| Gender |  |  |  |  |  |  |  |  |
| Male | 99.98 | 100 | 89.52 | 94.04 | 73.54 | 63.88 | 48.31 | 42.27 |
| Female | 99.98 | 99.94 | 93.85 | 84.58 | 72.08 | 72.96 | 47.4 | 36.38 |
| Cramer’s V | 0 | 0.02 | 0.08 | 0.15 | 0.02 | 0.10 | 0.01 | 0.06 |
| q-value | 1 | 0.138 | **＜0.001** | **＜0.001** | 0.167 | **＜0.001** | 0.476 | **＜0.001** |
| Age Group |  |  |  |  |  |  |  |  |
| Young Adult | 99.96 | 100 | 89.54 | 93.12 | 56.29 | 66.29 | 41.96 | 44.88 |
| Middle-Aged | 100 | 99.92 | 91.46 | 91.5 | 61.58 | 64.46 | 44.12 | 42.83 |
| Older Middle-Aged | 100 | 100 | 92.25 | 85.92 | 82.25 | 68 | 49.42 | 34.5 |
| Elderly | 99.96 | 99.96 | 93.5 | 86.71 | 91.12 | 74.92 | 55.92 | 35.08 |
| Cramer’s V | 0.01 | 0.02 | 0.05 | 0.10 | 0.32 | 0.09 | 0.11 | 0.09 |
| q-value | 0.654 | 0.413 | **＜0.001** | **＜0.001** | **＜0.001** | **＜0.001** | **＜0.001** | **＜0.001** |
| Region |  |  |  |  |  |  |  |  |
| Rural | 99.98 | 99.96 | 92.94 | 88.04 | 80.21 | 68.71 | 48.52 | 34.42 |
| Urban | 99.98 | 99.98 | 90.44 | 90.58 | 65.42 | 68.12 | 47.19 | 44.23 |
| Cramer’s V | 0 | 0.01 | 0.05 | 0.04 | 0.17 | 0.01 | 0.01 | 0.10 |
| q-value | 1 | 0.654 | **＜0.001** | **＜0.001** | **＜0.001** | 0.652 | 0.272 | **＜0.001** |
|  |  |  |  |  |  |  |  |  |
| Education |  |  |  |  |  |  |  |  |
| Low | 100 | 100 | 90.81 | 90.33 | 76.31 | 68.56 | 46.98 | 38.83 |
| High | 99.96 | 99.94 | 92.56 | 88.29 | 69.31 | 68.27 | 48.73 | 39.81 |
| Cramer’s V | 0.01 | 0.02 | 0.03 | 0.03 | 0.08 | 0 | 0.02 | 0.01 |
| q-value | 0.233 | 0.138 | **0.003** | **0.002** | **＜0.001** | 0.820 | 0.138 | 0.434 |
| Income Level |  |  |  |  |  |  |  |  |
| Low | 99.97 | 99.97 | 92.34 | 86.09 | 68.69 | 64.25 | 48.31 | 34.56 |
| Middle | 99.97 | 99.97 | 89.47 | 91.66 | 75.88 | 69.91 | 48.38 | 44.03 |
| High | 100 | 99.97 | 93.25 | 90.19 | 73.88 | 71.09 | 46.88 | 39.38 |
| Cramer’s V | 0.01 | 0 | 0.06 | 0.08 | 0.07 | 0.06 | 0.01 | 0.08 |
| q-value | 0.674 | 1 | **＜0.001** | **＜0.001** | **＜0.001** | **＜0.001** | 0.496 | **＜0.001** |

Note: Data are presented as the rate (%) of theme presence within each subgroup. q-values were derived from the Chi-squared test or Fisher's exact test, with p-values adjusted for multiple comparisons using the Benjamini-Hochberg (FDR) procedure. Statistical significance (q < 0.05) is highlighted in bold.

**Supplementary Table S2. Full Results of Multivariable Modified Poisson Regression Models for Thematic Presence**

| Theme | Predictor | Comparison | aRR (95% CI) | q-value |
| --- | --- | --- | --- | --- |
| Safety Management with Risk Control | Gender | Female vs. Male [Ref.] | 0.90 (0.89–0.91) | <.001 |
|  | Age Group | Middle-Aged vs. Young Adult [Ref.] | 0.98 (0.97–1.0) | 0.067 |
|  |  | Older Middle-Aged vs. Young Adult [Ref.] | 0.92 (0.91–0.94) | <.001 |
|  |  | Elderly vs. Young Adult [Ref.] | 0.93 (0.91–0.95) | <.001 |
|  | Region | Rural vs. Urban [Ref.] | 0.97 (0.96–0.99) | <.001 |
|  | Education | Low vs. High [Ref.] | 1.02 (1.01–1.04) | 0.002 |
|  | Income Level | Low vs. High [Ref.] | 0.95 (0.94–0.97) | <.001 |
|  |  | Middle vs. High [Ref.] | 1.02 (1.0–1.03) | 0.076 |
| Family Support | Gender | Female vs. Male [Ref.] | 0.98 (0.96–1.0) | 0.146 |
|  | Age Group | Middle-Aged vs. Young Adult [Ref.] | 1.09 (1.04–1.15) | <.001 |
|  |  | Older Middle-Agedd vs. Young Adult [Ref.] | 1.46 (1.41–1.52) | <.001 |
|  |  | Elderly vs. Young Adult [Ref.] | 1.62 (1.56–1.68) | <.001 |
|  | Region | Rural vs. Urban [Ref.] | 1.23 (1.2–1.26) | <.001 |
|  | Education | Low vs. High [Ref.] | 1.1 (1.08–1.13) | <.001 |
|  | Income Level | Low vs. High [Ref.] | 0.93 (0.9–0.96) | <.001 |
|  |  | Middle vs. High [Ref.] | 1.03 (1.0–1.05) | 0.092 |
| Technical Support and IV Management | Gender | Female vs. Male [Ref.] | 1.05 (1.04–1.06) | <.001 |
|  | Age Group | Middle-Aged vs. Young Adult [Ref.] | 1.02 (1.0–1.04) | 0.049 |
|  |  | Older Middle-Agedd vs. Young Adult [Ref.] | 1.03 (1.01–1.05) | 0.002 |
|  |  | Elderly vs. Young Adult [Ref.] | 1.04 (1.03–1.06) | <.001 |
|  | Region | Rural vs. Urban [Ref.] | 1.03 (1.02–1.04) | <.001 |
|  | Education | Low vs. High [Ref.] | 0.98 (0.97–0.99) | 0.004 |
|  | Income Level | Low vs. High [Ref.] | 0.99 (0.98–1.0) | 0.241 |
|  |  | Middle vs. High [Ref.] | 0.96 (0.95–0.97) | <.001 |
| Nurse Training and Event Analysis | Gender | Female vs. Male [Ref.] | 0.86 (0.82–0.9) | <.001 |
|  | Age Group | Middle-Aged vs. Young Adult [Ref.] | 0.95 (0.9–1.02) | 0.241 |
|  |  | Older Middle-Agedd vs. Young Adult [Ref.] | 0.77 (0.72–0.82) | <.001 |
|  |  | Elderly vs. Young Adult [Ref.] | 0.78 (0.73–0.84) | <.001 |
|  | Region | Rural vs. Urban [Ref.] | 0.78 (0.74–0.82) | <.001 |
|  | Education | Low vs. High [Ref.] | 0.98 (0.93–1.02) | 0.378 |
|  | Income Level | Low vs. High [Ref.] | 0.88 (0.82–0.94) | <.001 |
|  |  | Middle vs. High [Ref.] | 1.12 (1.06–1.18) | <.001 |
| Environmental Adjustment | Gender | Female vs. Male [Ref.] | 1.14 (1.11–1.17) | <.001 |
|  | Age Group | Middle-Aged vs. Young Adult [Ref.] | 0.97 (0.93–1.01) | 0.267 |
|  |  | Older Middle-Agedd vs. Young Adult [Ref.] | 1.03 (0.99–1.07) | 0.292 |
|  |  | Elderly vs. Young Adult [Ref.] | 1.13 (1.09–1.17) | <.001 |
|  | Region | Rural vs. Urban [Ref.] | 1.01 (0.98–1.04) | 0.611 |
|  | Education | Low vs. High [Ref.] | 1.0 (0.98–1.03) | 0.834 |
|  | Income Level | Low vs. High [Ref.] | 0.9 (0.87–0.93) | <.001 |
|  |  | Middle vs. High [Ref.] | 0.98 (0.95–1.01) | 0.378 |
| Pain and Medication Management | Gender | Female vs. Male [Ref.] | 0.98 (0.94–1.02) | 0.426 |
|  | Age Group | Middle-Aged vs. Young Adult [Ref.] | 1.05 (0.99–1.12) | 0.218 |
|  |  | Older Middle-Agedd vs. Young Adult [Ref.] | 1.18 (1.11–1.25) | <.001 |
|  |  | Elderly vs. Young Adult [Ref.] | 1.33 (1.26–1.41) | <.001 |
|  | Region | Rural vs. Urban [Ref.] | 1.03 (0.99–1.07) | 0.274 |
|  | Education | Low vs. High [Ref.] | 0.96 (0.92–1.0) | 0.146 |
|  | Income Level | Low vs. High [Ref.] | 1.03 (0.98–1.08) | 0.337 |
|  |  | Middle vs. High [Ref.] | 1.03 (0.98–1.09) | 0.316 |
| Communication and Education | Gender | Female vs. Male [Ref.] | 1.0 (1.0–1.0) | 1.0 |
|  | Age Group | Middle-Aged vs. Young Adult [Ref.] | 1.0 (1.0–1.0) | 0.378 |
|  |  | Older Middle-Agedd vs. Young Adult [Ref.] | 1.0 (1.0–1.0) | 0.378 |
|  |  | Elderly vs. Young Adult [Ref.] | 1.0 (1.0–1.0) | 1.0 |
|  | Region | Rural vs. Urban [Ref.] | 1.0 (1.0–1.0) | 1.0 |
|  | Education | Low vs. High [Ref.] | 1.0 (1.0–1.0) | 0.241 |
|  | Income Level | Low vs. High [Ref.] | 1.0 (1.0–1.0) | 0.378 |
|  |  | Middle vs. High [Ref.] | 1.0 (1.0–1.0) | 0.378 |
| Emotional Support and Stress Management and Education | Gender | Female vs. Male [Ref.] | 1.0 (1.0–1.0) | 0.146 |
|  | Age Group | Middle-Aged vs. Young Adult [Ref.] | 1.0 (1.0–1.0) | 0.241 |
|  |  | Older Middle-Aged vs. Young Adult [Ref.] | 1.0 (1.0–1.0) | 1.0 |
|  |  | Elderly vs. Young Adult [Ref.] | 1.0 (1.0–1.0) | 0.378 |
|  | Region | Rural vs. Urban [Ref.] | 1.0 (1.0–1.0) | 0.633 |
|  | Education | Low vs. High [Ref.] | 1.0 (1.0–1.0) | 0.146 |
|  | Income Level | Low vs. High [Ref.] | 1.0 (1.0–1.0) | 1.0 |
|  |  | Middle vs. High [Ref.] | 1.0 (1.0–1.0) | 1.0 |

aRR = adjusted Relative Risk; CI = Confidence Interval; Ref. = Reference group.

**Supplementary Table S3 Sociodemographic Characteristics of the Expert Review Subsample and Descriptive Statistics of Quality Scores (n = 500)**

**Panel A. Sociodemographic Characteristics**

| Characteristic | n (%) |
| --- | --- |
| Gender |  |
| Male | 253 (50.6) |
| Female | 247 (49.4) |
| Age group |  |
| Young Adult | 124 (24.8) |
| Middle-Aged | 125 (25.0) |
| Older Middle-Aged | 125 (25.0) |
| Elderly | 126 (25.2) |
| Region |  |
| Urban | 260 (52.0) |
| Rural | 240 (48.0) |
| Education |  |
| High education | 252 (50.4) |
| Low education | 248 (49.6) |
| Income level |  |
| High income | 175 (35.0) |
| Middle income | 165 (33.0) |
| Low income | 160 (32.0) |

**Panel B. Descriptive Statistics of Expert-Rated Quality Scores**

| Dimension | Mean | SD | Median | Min | Max | Normality test (p) |
| --- | --- | --- | --- | --- | --- | --- |
| Safety | 4.55 | 0.47 | 4.5 | 3.5 | 5.0 | < .001 |
| Clinical Applicability | 4.37 | 0.46 | 4.0 | 3.5 | 5.0 | < .001 |
| Completeness | 4.49 | 0.48 | 4.5 | 3.5 | 5.0 | < .001 |
| Overall mean | 4.47 | 0.26 | 4.5 | 4.0 | 5.0 | < .001 |
